# Left ventricular unloading therapy using Impella 5.5 after emergency surgery for acute myocardial infarction mechanical complication: a case report

**DOI:** 10.1186/s13019-024-02879-5

**Published:** 2024-06-26

**Authors:** Kenji Iino, Yoshitaka Yamamoto, Hideyasu Ueda, Hirofumi Takemura

**Affiliations:** Department of Cardiovascular Surgery, Kanazawa University, 13-1 Takaramachi, Kanazawa, 920-8641 Japan

**Keywords:** Impella, Acute myocardial infarction, Ventricular septal rupture, Left ventricular free wall rupture.

## Abstract

**Background:**

Following an acute myocardial infarction (AMI), surgery for left ventricular free wall rupture (LVFWR) and ventricular septal rupture (VSR) has a high in-hospital mortality rate, which has not improved significantly over time. Unloading the LV is critical to preventing excessive stress on the repair site and avoiding problems such as bleeding, leaks, patch dehiscence, and recurrence of LVFWR and VSR because the tissue is so fragile. We present two cases of patients who used Impella 5.5 for LV unloading following emergency surgery for AMI mechanical complications.

**Case presentation:**

A 76-year-old male STEMI patient underwent fibrinolysis of the distal right coronary artery. Three days later, he passed out and went into shock. Echocardiography revealed a cardiac tamponade. We found an oozing-type LVFWR on the posterolateral wall and treated it with a non-suture technique using TachoSil. Before the patient was taken off CPB, Impella 5.5 was inserted into the LV via a 10 mm synthetic graft connected to the right axillary artery. We kept the flow rate above 4.0 to 4.5 L/min until POD 3 to reduce LV wall tension while minimizing pulsatility. On POD 6, we weaned the patient from Impella 5.5. A postoperative cardiac CT scan showed no contrast leakage from the LV. However, a cerebral hemorrhage on POD 4 during heparin administration complicated his hospitalization. Case 2: A diagnosis of cardiogenic shock caused by STEMI occurred in an 84-year-old male patient, who underwent PCI of the LAD with IABP support. Three days after PCI, echocardiography revealed VSR, and the patient underwent emergency VSR repair with two separate patches and BioGlue applied to the suture line between them. Before weaning from CPB, we implanted Impella 5.5 in the LV and added venoarterial extracorporeal membrane oxygenation (VA-ECMO) support for right heart failure. The postoperative echocardiography revealed no residual shunt.

**Conclusions:**

Patients undergoing emergency surgery for mechanical complications of AMI may find Impella 5.5 to be an effective tool for LV unloading. The use of VA-ECMO in conjunction with Impella may be an effective strategy for managing VSR associated with concurrent right-sided heart failure.

**Supplementary Information:**

The online version contains supplementary material available at 10.1186/s13019-024-02879-5.

## Background

Left ventricular free wall rupture (LVFWR), ventricular septal rupture (VSR), and papillary muscle rupture (PMR) after acute myocardial infarction (AMI) are potentially fatal pathologies. Cardiac surgery for these mechanical complications of AMI has a high in-hospital mortality rate, which has not improved significantly over time [1–3]. Left ventricular (LV) unloading is essential to avoid excessive tension on the repair site after emergency surgery, which leads to burdensome hemostasis of the LV wall, a residual leak, patch dehiscence, and recurrence of LVFWR and VSR. Venoarterial extracorporeal membrane oxygenation (VA-ECMO) used after surgery provides circulatory support; however, in the context of poor LV function, the resultant increase in LV afterload causes elevated LV end-diastolic pressure [4]. Impella 5.5 (Abiomed, Danvers, MA, USA) can provide adequate LV unloading and hemodynamic support [4]. We present two cases using Impella 5.5 for LV unloading after emergency surgery for an AMI mechanical complication.

## Case Presentation

### Case 1

**Fig. 1:**
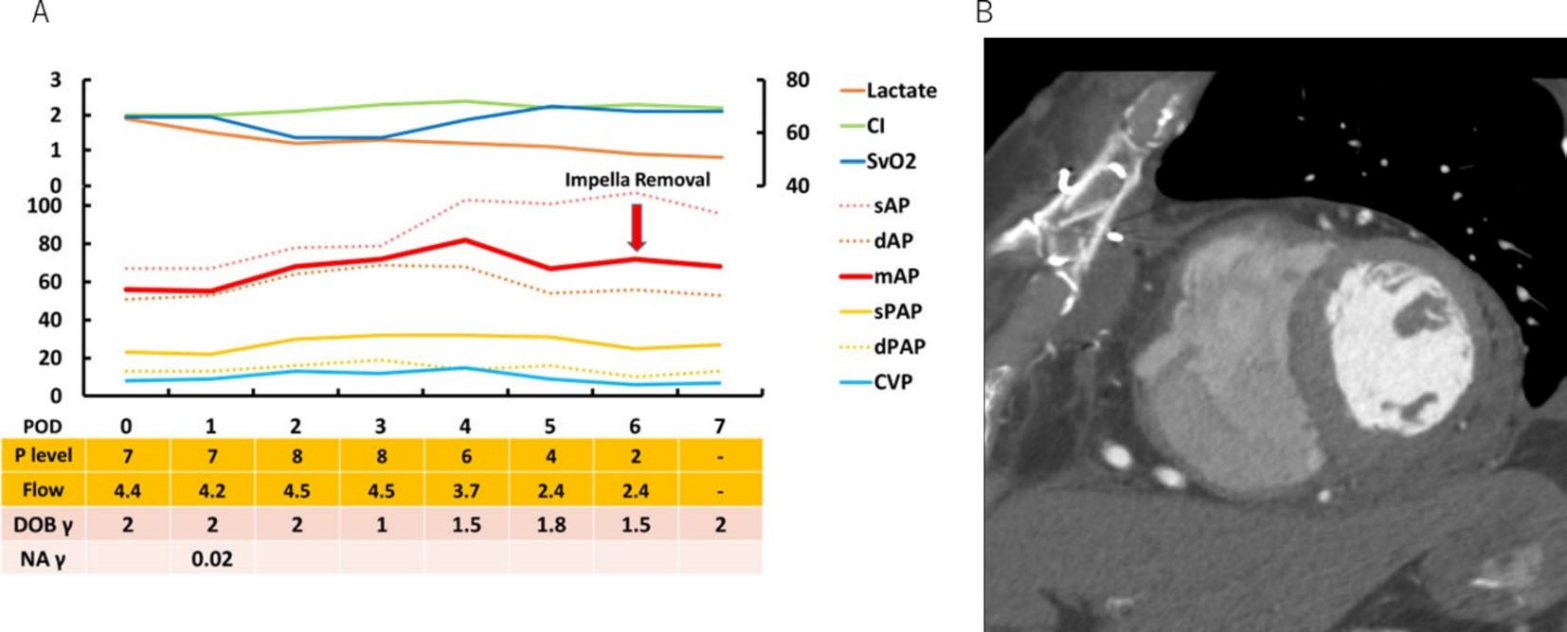
Clinical progress (**A**) and postoperative echo findings (**B**) in case 1. Clinical progress (**A**) and postoperative computed tomography showing repaired LVFWR without contrast leakage from the ventricle (**B**). SAP, systolic arterial pressure; dAP, diastolic arterial pressure; CI, cardiac index; mAP, mean arterial pressure; SvO2, mixed venous oxygen saturation; CVP, central venous pressure; sPAP, systolic pulmonary artery pressure; dPAP, diastolic pulmonary artery pressure; Flow, estimated blood flow with Impella; POD, postoperative day; P level, support level of Impella; DOB, dobutamine; NA, noradrenaline

A 76-year-old man was diagnosed with ST-elevation myocardial infarction (STEMI) and received fibrinolytic therapy for distal right coronary artery occlusion at another hospital. The patient did not have a rescue PCI or interventional procedure following thrombolytic therapy. Three days after fibrinolytic therapy, the patient fell into shock due to cardiac tamponade. He was transported to our hospital for an urgent surgery. On arrival, his hemodynamics had deteriorated (heart rate: 115 min^− 1^, systolic arterial pressure: 50 mmHg, and diastolic arterial pressure: 38 mmHg). Laboratory data demonstrated serum aspartate aminotransferase (357 IU/L), alanine aminotransferase (217 IU/L), creatinine (1.07 mg/dL), lactate (4.0 mmol/L), NT-pro brain natriuretic peptide (BNP) (2247 pg/ml), and troponin T (1.470 ng/mL). The blood gas analysis revealed pH 7.468, pCO2 23.6 mmHg, pO2 72.4 mmHg, HCO3 16.8 mmol/L, and BE -5.4 mmol/L. He had received EVER treatment for an abdominal aortic aneurysm eight years ago at another hospital. A type 2 endoleak persisted, and the abdominal aortic aneurysm expanded. Therefore, the placement of IABP or peripheral VA-ECMO was not feasible. He was transferred to the OR using vasopressor administration (norepinephrine: 0.17 µg kg^− 1^min^− 1^). Cardiopulmonary bypass (CPB) was initiated via the right femoral vein and left axillary artery before median sternotomy. The posterolateral wall was found to have oozing-type LVFWR, and it was treated without sutures using TachoSil and fibrin glue. Because the repair site was extremely fragile, we decided to initiate the Impella 5.5 device to ensure sufficient LV unloading postoperatively. Impella 5.5 was inserted into the LV via a 10-mm synthetic graft anastomosed to the right axillary artery before weaning off CPB (Video). Up to postoperative day (POD) 3, the flow rate of Impella was maintained above 4.0 to 4.5 L/min (P7 or P8) to reduce LV wall tension with a minimum amount of pulsatility. Although hemodynamics was steady, POD 4 showed anisocoria. A brain computed tomography (CT) scan revealed severe cerebral hemorrhage. The patient then underwent endoscopic hematoma evacuation and external ventricular drainage. On POD 6, he was weaned from Impella (Fig. 1A). On POD 25, a cardiac CT scan revealed no contrast leakage from the LV (Fig. 1B). He had a tracheotomy on POD 26 and required ventilator support throughout his hospital stay. His hospital course was complicated by a severe cerebral hemorrhage that had occurred before weaning from Impella and led to his death on POD 68.

## Case 2

**Fig. 2:**
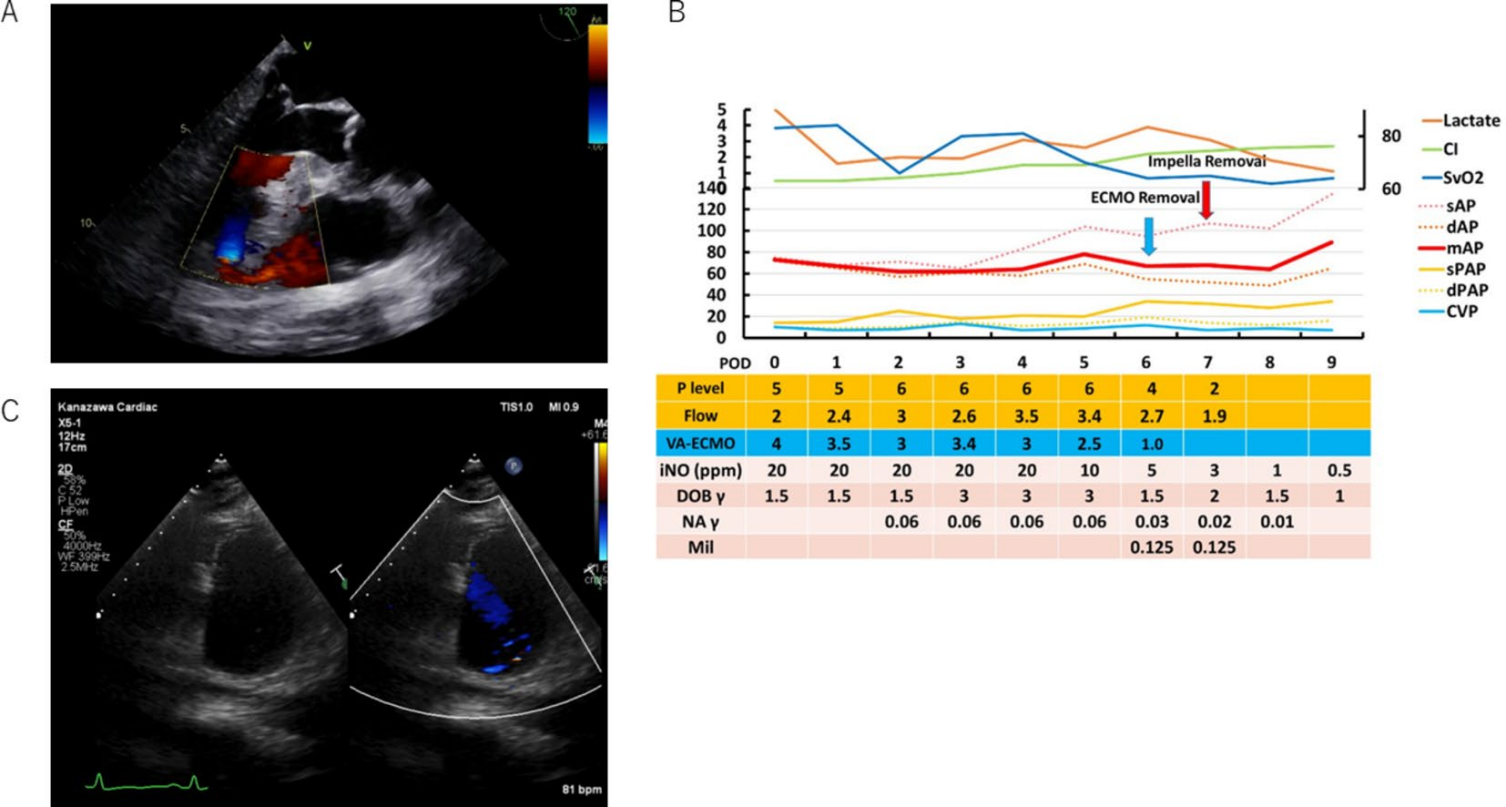
Preoperative echo findings (**A**) Clinical progress (**B**) and postoperative echo findings (**C**) in case 2. Transesophageal echocardiography showing an anterior VSR (**A**), Clinical progress (**B**) and postoperative echocardiography showing a repaired VSR without a residual shunt (**C**). SAP, systolic arterial pressure; dAP, diastolic arterial pressure; CI, cardiac index; mAP, mean arterial pressure; SvO2, mixed venous oxygen saturation; CVP, central venous pressure; sPAP, systolic pulmonary artery pressure; dPAP, diastolic pulmonary artery pressure; Flow, estimated blood flow with Impella; POD, postoperative day; P level, support level of Impella; VA-ECMO, venoarterial extracorporeal membrane oxygenation flow; iNO, inhaled nitric oxide; DOB, dobutamine; NA, noradrenaline; Mil, milrinone

An 84-year-old man was diagnosed with cardiogenic shock because of STEMI and underwent percutaneous coronary intervention (PCI) to the left anterior descending artery with intra-aortic balloon pump (IABP) support at another hospital. Three days after PCI, echocardiography revealed a VSR. The patient was transferred to our hospital for emergency surgery. On arrival, his systolic blood pressure was 97, heart rate was 135 bpm, and oxygen saturation was 96% with 6 L/min oxygen supply. Ventricular function was impaired with an ejection fraction of 40% with dobutamine support (10 µg kg^− 1^min^− 1^) and IABP support. Blood tests revealed elevated troponin T concentration (10.85 ng/mL), NT-pro BNP (41,480 pg/mL), and creatinine (3.39 mg/dL). Transesophageal echocardiography revealed an anterior VSR (Fig. 2A). We repaired the VSR using the two-patch technique with BioGlue [5]. During weaning off CPB, the patient had biventricular failure (cardiac index of 1.0 L/min/m^2^, pulmonary capillary wedge pressure (PCWP) 22 mmHg, and pulmonary artery pulsatility index of 0.4). Before weaning off CPB, Impella 5.5 was inserted into the left ventricle and IABP was removed. Thereafter, venoarterial extracorporeal membrane oxygenation (VA-ECMO) was initiated via the right femoral artery and vein and inhaled nitric oxide (iNO) administration was initiated for right-sided heart failure (video). The operating time was 340 min. The cross-clamp was completed in 105 min. The CPB took 235 min. The flow rate of ECMO was maintained between 3.0 and 4.0 L/min, and the Impella was maintained at P5 or P6 level until POD 4 to reduce tension on the sutured patches and the LV wall while maintaining minimal pulsatility. ECMO, Impella, and iNO were weaned off on PODs 6, 7, and 10, respectively (Fig. 2B). He was extubated on POD 23. The LV ejection fraction following surgery was 39%, with an end-diastolic dimension of 49 mm and an end-systolic dimension of 40 mm. The estimated right ventricular systolic pressure was 30 mmHg, with no residual shunt (Fig. 2C). During his rehabilitation in the general ward, he was infected with COVID-19 and underwent treatment with remdesivir and dexamethasone for COVID-19 pneumonia. On POD 80, the patient was transferred to the previous hospital for further rehabilitation. Informed consent was provided by the patient’s families.

## Discussion and conclusions

The major concern regarding sutureless repair for LVFWR is the high incidence of postoperative rerupture, which is associated with high mortality. Okumura et al. reported the results of 35 patients undergoing sutureless repair for LVFWR [6]. The rerupture rates were 12% in patients with the oozing-type LVFWR. However, in cases of VSR resulting from AMI, despite improvements in surgical technique, residual leakage, uncontrolled bleeding, low cardiac output, technical difficulties, higher mortality, and recurrence of VSR after VSR repair still occur. These findings indicate that effective LV unloading after surgical repair is essential to prevent the occurrence of LVFWR and VSR recurrence in patients with AMI mechanical complications. In this context, the objective of hemodynamic support is to simultaneously decrease the afterload and enhance systemic blood pressure and organ perfusion, while concurrently reducing LV end-diastolic pressure and PCWP.

IABP has been widely employed for LV unloading after cardiac surgery. However, the effectiveness of IABP remains debatable [7, 8]. According to recent research, Impella provides better LV unloading than IABP [9, 10]. Impella 5.5 achieves a higher pump flow rate than prior percutaneous devices. This higher pump flow translates to more effective unloading of the left ventricle, and the design of Impella 5.5 without the pigtail tip is friendly to the fragile LV wall due to infarction. In patients with left heart failure, Impella offers effective hemodynamic support. With an increase in the pump flow rate, there is progressive unloading of the left ventricle, as evidenced by a leftward shift in the pressure-volume loop (PVL). This leads to a reduction in the peak LV pressure. In addition, there is a significant decrease in both pressure-volume area (PVA) and myocardial oxygen consumption (MVO2). Concurrently, arterial pressure rises, resulting in an increasing disparity between peak LV pressure and arterial pressure [7]. However, in patients with concomitant right heart failure, Impella cannot have adequate preload, resulting in unsteady flow from Impella and insufficient hemodynamic support. In such cases, VA-ECMO can provide end-organ perfusion with retrograde aortic flow. However, it may result in a rightward shift of PVL, increase in afterload, and subsequent rise in end-diastolic and end-systolic volumes and elevation in PCWP [4]. Effective hemodynamic support in the setting of biventricular failure requires VA-ECMO with concurrent Impella support for afterload reduction. At our facility, we recommend Impella for left heart failure with a PCWP of ≥ 15, and VA-ECMO for right heart failure with a Pulmonary Artery Pulsatility Index (PAPi) of < 1.

Case 1 involved a patient in shock with a possible blow-out type of FWR. The post-EVER abdominal aortic aneurysm prevented the placement of IABP and peripheral VA-ECMO, necessitating the immediate start of cardiopulmonary bypass prior to median sternotomy. Due to the post-EVER abdominal aortic aneurysm, we chose the axillary artery over the femoral artery as the cannulation site. We also intended to place the Impella intraoperatively before weaning the patient off the CPB prior to surgery. Eulert-Grehn et al. found that a single arterial access technique was effective for connecting the bifurcated vascular graft to the right axillary artery for the CPB Impella and arterial cannulation [11]. However, we do not possess a 10 mm branched vascular graft. Therefore, to perform this technique, we would need time to anastomose the side branch to the graft, resulting in a branched graft, as well as sufficient time to anastomose the branched graft to the axillary artery.

However, we cannulate the axillary artery for the CPB in a more straightforward and time-efficient manner, because we use the Seldinger method. Therefore, to hasten the establishment of the CPB, we cannulated the left axillary artery directly. Then, following FWR repair, we anastomosed a straight vascular graft to the right axillary artery beneath the CPB, implanted the Impella, and weaned the CPB. The patient did not have the right-sided heart failure. To reduce the LV wall tension with little pulsatility, an Impella 5.5 flow rate of ˃4.5 L/min may be maintained until POD 3. This resulted in a notable drop in the LV pressure and volume, which could prevent the rerupture of the LV wall. However, the patient experienced a cerebral hemorrhage with ventricular perforation in the right temporal lobe on the POD4. Hassett et al. reported that in a prospective study of 79 patients with Impella insertions, 6 (7.6%) had strokes at a median of 5 days (range 1–8 days), with 2 ofthem having intracerebral hemorrhage. They attributed the intracerebral hemorrhage to anticoagulant therapy and thrombocytopenia [12]. Our protocol for anticoagulation for Impella after open heart surgery is: no heparin in the purge solution until 6 h postoperatively, 2500 units from 6 to 24 h, 5000 units from 24 to 48 h, and 10,000 units for ≥ 48 h. We then begin systemically with 500 units of heparin per hour and adjust the heparin dose to achieve an activated clotting time (ACT) of 150–180 s and an activated partial thromboplastin time (aPTT) of 50–70 s.

We measure the ACT every 6 h, the APTT every 24 h, and adjust the heparin dose by increasing or decreasing 100 units per hour every 6 h to achieve the desired results.

By POD2, the patient’s purge solution had 10,000 units of heparin. From POD3, the patient was given 500 units of heparin per hour systemically. After 6 h of systemic heparin administration at 12,000 units per day, the ACT was ˃150 s. At the onset of cerebral hemorrhage, we properly managed ACT, and platelet levels were within reference limits. There was no known causal relationship between the development of cerebral hemorrhage and Impella use.

In case 2, because of right heart failure, VA-ECMO was required to maintain end-organ perfusion, which led to the resultant increase in LV afterload. The combination of iNO and Impella 5.5 was effectively maintained a steady flow from Impella and LV unloading.

When combined with Impella for heart recovery, we typically withdraw VA-ECMO first. Our criteria for withdrawing from VA-ECMO are CVP < 15 mmHg, PAPI ˃than 1, mean aortic blood pressure ˃65 mmHg, and mixed venous saturation (SvO2) ˃60%.VA-ECMO flow is gradually reduced to 1.0 L while the above conditions are monitored. If the conditions are met and dobutamine (DOB) is < 3 µg/kg/min, the patient will be weaned off VA-ECMO. Our criteria for Impella withdrawal are PCWP < 15 mmHg, cardiac power output ˃0.6, and DOB < 3 µg/kg/min.

ECMO and Impella were successfully weaned from this patient on PODs 6 and 7, respectively. We found no evidence of LV aneurysm development or residual VSR.

Instead of VA-ECMO, a temporary right ventricular assist device (RVAD) with percutaneous cannulation would be preferable for patients whose RV function is impaired. This would allow for early ventilator removal, early extubation, and rehabilitation [13].

However, this procedure requires the use of a special cannula to pulmonary artery via the femoral vein or right internal jugular vein. Unfortunately, these specialized cannulas are unavailable in Japan. Traditional RVAD allows for full right heart support followed by weaning, but it is often associated with more invasive surgery, the need for an extra thoracotomy for decannulation, limited mobility after surgery, and/or longer retention of prosthetic material in situ (such as a prosthetic graft anastomosed to the pulmonary artery and tunneled intercostally).Consequently, because VA-ECMO enables cannula removal at the bedside, we selected it over traditional RVAD.

Patients undergoing emergency surgery for mechanical complications caused by AMI may find the Impella 5.5 to be a useful tool for LV unloading. Using VA-ECMO in conjunction with Impella might be a beneficial strategy for managing VSR associated with simultaneous right-sided heart failure.Table 1

**Table 1:** Timeline

Patient 1	
8 years prior to event	EVER treatment for an abdominal aortic aneurysm
3 days prior to event	He received fibrinolytic therapy for STEMI with distal right coronary artery occlusion.
Day 0	He fell into shock due to cardiac tamponade. Oozing-type LVFWR was treated without sutures using TachoSil and fibrin glue.
POD 4	Cerebral hemorrhage.
POD 6	Weaning from Impella.
POD 26	Tracheotomy.
POD 68	Died.
**Patient 2**	
3 days prior to event	He underwent percutaneous coronary intervention to the left anterior descending artery because of STEMI
Day 0	He underwent VSR repair.
POD 6	Weaning from ECMO.
POD 7	Weaning from Impella.
POD 10	Weaning from iNO.
POD 23	Extubation.
POD 80	He was transferred to the previous hospital for further rehabilitation

### Electronic supplementary material

Below is the link to the electronic supplementary material.

Supplementary Material 1

## Data Availability

No datasets were generated or analysed during the current study.

## Abbreviations

AMI: Acute Myocardial Infarction
CPB: Cardiopulmonary Bypass
CT: Computed Tomography
CVP: Central Venous Pressure
DOB: Dobutamine
iNO: Inhaled Nitric Oxide
IABP: Intra-Aortic Balloon Pump
LV: Left Ventricular, Left Ventricle
LVEF: Left Ventricular Ejection Fraction
LVFWR: Left Ventricular Free Wall Rupture
MVO2: Myocardial Oxygen Consumption
PAPi: Pulmonary Artery Pulsatility Index
PCI: Percutaneous Coronary Intervention
PCWP: Pulmonary Capillary Wedge Pressure
PMR: Papillary Muscle Rupture
POD: Postoperative Day
PVA: Pressure-Volume Area
PVL: Pressure-Volume Loop
RVAD: Right Ventricular Assist Device
STEMI: ST-Elevation Myocardial Infarction
SvO2: Mixed Venous Saturation
VA-ECMO: Venoarterial Extracorporeal Membrane Oxygenation
VSR: Ventricular Septal Rupture
